# Application of HPLC-DAD for In Vitro Investigation of Acetylcholinesterase Inhibition Activity of Selected Isoquinoline Alkaloids from *Sanguinaria canadensis* Extracts

**DOI:** 10.3390/molecules26010230

**Published:** 2021-01-05

**Authors:** Tomasz Tuzimski, Anna Petruczynik

**Affiliations:** 1 Department of Physical Chemistry, Medical University of Lublin, Chodźki 4a, 20-093 Lublin, Poland; 2 Department of Inorganic Chemistry, Medical University of Lublin, Chodźki 4a, 20-093 Lublin, Poland

**Keywords:** acetylcholinesterase activity inhibition, isoquinoline alkaloids, *Sanguinaria canadensis*, HPLC-DAD

## Abstract

Isoquinoline alkaloids may have a wide range of pharmacological activities. Some of them have acetylcholinesterase activity inhibition. Nowadays, neurodegenerative disorders such as Alzheimer’s disease have become a serious public health problem. Searching for new effective compounds with inhibited acetylcholinesterase activity is one of the most significant challenges of modern scientific research. The aim of this study was the in vitro investigation of acetylcholinesterase activity inhibition of extracts obtained from *Sanguinaria canadensis* collected before, during and after flowering. The acetylcholinesterase activity inhibition of these extracts has not been previously tested. The aim was also to quantify selected alkaloids in the investigated extracts by high performance liquid chromatography (HPLC). The analyses of alkaloid content were performed using HPLC in reversed phase (RP) mode using Polar RP column and mobile phase containing acetonitrile, water and ionic liquid (IL). The acetylcholinesterase activity inhibition of the tested plant extracts and respective alkaloid standards were examined using high performance liquid chromatography with diode-array detector (HPLC-DAD) for the quantification of 5-thio-2-nitro-benzoic acid, which is the product of the reaction between the thiocholine (product of the hydrolysis of acetylthiocholine reaction) with Ellman reagent. The application of the HPLC method allowed for elimination of absorption of interfering components, for example, alkaloids such as sanguinarine and berberine. It is revealed that the HPLC method can be successfully used for the evaluation of the acetylcholinesterase inhibitory activity in samples such as plant extracts, especially those containing colored components adsorbing at wavelength in the range 405–412 nm. The acetylcholinesterase inhibition activity synergy of pairs of alkaloid standards and mixture of all investigated alkaloids was also determined. Most investigated alkaloids and all *Sanguinaria canadensis* extracts exhibited very high acetylcholinesterase activity inhibition. IC_50_ values obtained for alkaloid standards were from 0.36 for berberine to 23.13 µg/mL for protopine and from 61.24 to 89.14 µg/mL for *Sanguinaria canadensis* extracts. Our investigations demonstrated that these plant extracts can be recommended for further in vivo experiments to confirm their acetylcholinesterase activity inhibition.

## 1. Introduction

Acetylcholinesterase enzymes have drawn attention since they can be a therapeutic objective for the treatment of Alzheimer’s disease. Alzheimer’s disease is an irreversible progressive neurodegenerative disorder of the central nervous system, which is described by a progressive loss of cognitive abilities [1]. This disease is characterized by a loss of cholinergic neurons in the brain and is associated with decreased levels of acetylcholine [2]. One of the major approved therapies for Alzheimer’s disease is based on a reduction of the cognitive deficits by enhancing cholinergic transmission through inhibition of acetylcholinesterase [3,4]. This is achieved by the use of cholinesterase inhibitors, which inhibit the enzyme responsible for breaking down acetylcholine. Cholinesterase play an important role in the regulation of neurotransmission by catalyzing the degradation of neurotransmitters. The enzyme hydrolyzes the active neurotransmitter acetylcholine into the inactive compounds choline and acetic acid. Currently, against Alzheimer’s disease, drug research and development are based on the cholinergic hypothesis that supports the cognition improvement by the regulation of the synthesis and release of acetylcholine in the brain [5].

The acetylcholinesterase inhibitory activity test is one of the important areas of natural product research. Plants are an important reservoir of bioactive specialized metabolites including acetylcholinesterase inhibitors. Therefore, the empirical approach to discover new drugs from the systematic screening of plant extracts or plant-derived substances coupled with ethnopharmacological knowledge still remains an important strategy for finding new biologically active compounds.

Both in vivo and in vitro methods are currently used to assess the acetylcholinesterase inhibition activity of compounds. In vivo screening methods are time consuming, entail significant human and material resources, and usually require large samples. In vitro methods are usually fast and require small samples, and therefore, are suitable for analyzing of active compounds, especially in plants. Among plant-derived cholinesterase inhibitors, alkaloids are regarded as the strongest acetylcholinesterase inhibitors. This activity is exhibited by some alkaloids, for example, Amarylidaceae alkaloids [5,6,7], isoquinoline alkaloids [5,8], and indole alkaloids [9].

Many plant extracts demonstrate acetylcholinesterase activity, for example, extract from *Crinum jagus* containing Amaryllidaceae and isoquinolinone alkaloids [6], *Cryptocarya* species containing isoquinoline alkaloids [10], *Narcissus pseudonarcissus* L. containing Amarylidaceae alkaloids [7], *Catharanthus roseus* containing indole alkaloids [9], seco-phenanthroindolizidine and proaporphine alkaloids from *Cryptocarya densiflora* Blume [10], *Ocotea percoriacea* containing benzylisoquinoline, bisbenzylisoquinoline, aporphine, proaporphine, phenanthrene, and morphinane alkaloids [9], *Rauvolfia vomitoria* containing monoterpene indole alkaloids [11], *Crinum jagus* containing Amarylidaceae and isoquinoline alkaloids [6], *Lycoris longituba* containing Amaryllidaceae alkaloid [12], *Psychotria leiocarpa* containing alkaloid vincosamide [13], *Huperzia serrata* containing macrocyclic *Lycopodium* alkaloids [14], *Psychotria nemorosa* containing azepine-indole alkaloids [15], *Uncaria rhynchophylla* containing indole alkaloids [16], *Berberis vulgaris* containing isoquinoline alkaloids [17], *Lycopodium platyrhizoma* containing *Lycopodium* alkaloids [18], *Huperzia cunninghamioides* containing *Lycopodium* alkaloids [19], *Corydalis mucronifera* containing isoquinoline alkaloids [20], *Palicourea sessilis* containing indole alkaloids [21], and *Zephyranthes candida* containing galanthamine, plicamine, and secoplicamine [22]. There have also been published articles summarizing the application of plant materials containing alkaloids for inhibition of acetylcholinesterase activity [23,24].

Extracts obtained for *Sanguinaria canadensis* L. are used in herbal medicines due to their anti-bacterial, tumoricidal, antioxidant, and immunomodulatory properties [25]. Benzophenanthridine alkaloids are believed to be the primary bioactive components in Sanguinaria spp, including sanguinarine, chelirubine, chelerythrine, and other alkaloids [25].

Acetylcholinesterase in vitro inhibition was investigated by spectrophotometry [6,7,9,12,14,16,17,18,20,22,26,27,28,29,30]. Usually acetylcholinesterase activity was determined according to Ellman et al. [31]. In their method the kinetics of acetylthiocholine iodide hydrolysis by acetylcholinesterase were measured spectrophotometrically. Currently, measurements are most often carried out with the application of 96-well microplates [7,9,12,15,21]. 

Chromatography was rarely applied for determination of acetylcholinesterase activity inhibition. Methods based on thin layer chromatography (TLC)-autography were sometimes applied for this purpose [32,33]. Wang et al. developed the HPLC method for determination of acetylcholinesterase inhibitory activity [34]. The determination of the acetylcholinesterase inhibitory activity of anthocyanin in blueberry extract and purple potato extract was performed on octadecyl (C18) column with mobile phase containing methanol, water, and trimethylamine. The chromatography method has high sensitivity, selectivity, and wide application, and additionally rules out the color interference caused by changes in pH. The results obtained by the authors showed that the inhibition ratios obtained from spectrophotometry were similar and approximately lower than 10% and the values of absorbance mostly exceed 0.8, but the absorbance in the range of 0.15–0.8 to should guarantee smaller relative error for spectrophotometry. While the inhibition ratios gained from the HPLC method were higher than 20% and had greater difference, they had better comparability to the various concentrations of anthocyanin and its extracts.

The chromatographic method is especially suitable for determination of acetylcholinesterase activity in samples, for example, some plant extracts containing the components which exhibit adsorption at about 412 nm. These samples also exhibit adsorption at the same wavelength as 5-thio-2-nitro-benzoic acid and, if the spectrophotometric method is used, they may cause errors in its quantification, while in the chromatographic method the individual components of the sample are separated which enables determination of only 5-thio-2-nitro-benzoic acid.

In this paper an HPLC method using a phenyl column and mobile phase containing addition of ionic liquid for separation of some isoquinoline alkaloids collected before, during, and after flowering was described. We also evaluate the acetylcholinesterase inhibition of *Sanguinaria canadensis* plant extracts by chromatographic method.

In our investigations for determination of the inhibition of acetylcholinesterase activity, the HPLC-DAD method was applied, which allows for a much more accurate determination of this activity compared to the commonly used spectrophotometric method. The use of chromatography to determine the inhibition of acetylcholinesterase activity for the quantification of 5-thio-2-nitro-benzoic acid, which is the product of the reaction between the thiocholine (product of the hydrolysis of acetylthiocholine reaction) with Ellman reagent, while it eliminates the measurement of adsorption at the same wavelength from other components, for example, alkaloids such as sanguinarine and berberine.

## 2. Results and Discussion

### 2.1. Determination of Alkaloid Contents in Plant Extracts

Alkaloid standards were chromatographed on Polar RP 8 column in eluent system containing acetonitrile, water, and 0.04 mL^−1^ of 1-butyl-3-methylimidazolium tetrafluoroborate in gradient system described in the Section 3. The chromatographic condition was based on the previously published method applied for determination of isoquinoline alkaloids after appropriate modification [35,36]. The Limits of quantification (LOQs) for most investigated alkaloids were determined in previous study and were 0.0457 mg/mL for berberine, 0.0123 mg/mL for chelerythrine, 0.0288 mg/mL for protopine, and 0.0371 mg/mL for sanguinarine [36]. For chelidonine, LOQ = 0.0336 mg/mL was determined.

**Figure 1:**
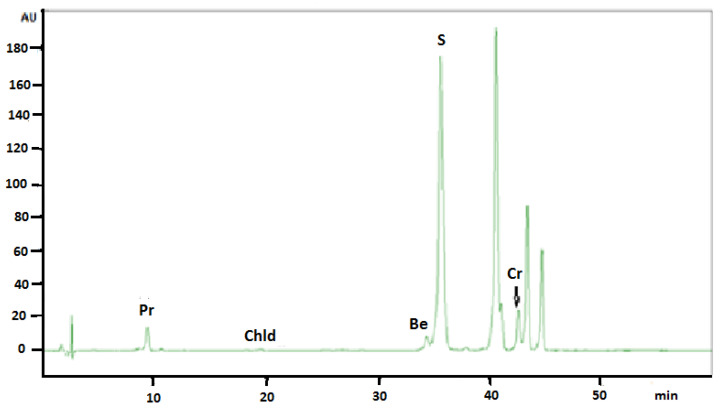
HPLC-DAD chromatogram obtained from *Sanguinaria canadensis* extract collected during flowering. Be: Berberine; Cr: Chelerythrine; Chld: Chelidonine; Pr: Protopine; S: Sanguinarine.

The same chromatographic conditions were developed for quantitative determination of selected isoquinoline alkaloids in plant extracts. The identities of the analyte peaks in plant extracts were confirmed by the comparison of their retention times and UV spectra with the retention times and spectra of alkaloid standards. The application of this chromatographic system allowed obtaining of very symmetrical peaks, high system efficiency, good analytes separation selectivity, and their separation from the matrix. Figure 1 presents typical chromatogram obtained from *Sanguinaria canadensis* extract collected during flowering.

Retention times of alkaloid standards are presented in Table 1.

The quantitative analysis of alkaloids in plant extracts was performed by a calibration curve method. The number of replicates was three for all concentration of all alkaloids. The content of alkaloids in extracts obtained from *Sanguinaria canadensis* collected before flowering, during flowering, and after flowering was determined. From 1 g of dry plant materials collected before, during, and after plant flowering, 12.58 mg, 15.64 mg, and 14.95 mg of dry extracts were obtained, respectively. The number of replicates was three for all plant materials. Significant differences were found in the average content of alkaloids in extracts obtained from plants collected before, during, and after flowering (Table 1). Isoquinoline alkaloids berberine, chelerythrine, chelidonine, protopine, and sanguinarine were determined in extracts obtained from *Sanguinaria canadensis*. A very high content of sanguinarine and chelerythrine was found in all investigated extracts. The content of sanguinarine, was the lowest before flowering (about 4.85 mg/g of plant material), it almost doubled during the flowering of the plant (about 9.59 mg/g of plant material), and decreased again after flowering (about 6.92 mg/g of plant material). High contents of chelerythrine have also been found in investigated plant extracts. The lowest concentration of chelerythrine, similar to sanguinarine, was found in the extract obtained from *Sanguinaria canadensis* before flowering (2.72 mg/g of plant material). Almost twice the concentration of this alkaloid was observed in the extract obtained from this plant during flowering, while the highest content of chelerythrine in the extract obtained after flowering was determined (6.92 mg/g of plant material). The content of the other quantified alkaloids was much lower, but also changed with the stage of plant vegetation. For example, in extracts from *Sanguinaria Canadensis* collected before flowering and during flowering, protopine was detected below LOQ value and increased to 0.1075 mg/g of plant material before flowering. Chelidonine was identified in small amounts only during this plant flowering (<LOQ). The lowest amount of all investigated alkaloids was found in the extract obtained from the plant before flowering. For some alkaloids (berberine, chelidonine, and sanquinarine) the content of alkaloids increased significantly during plant flowering and slightly decreased after flowering, while the content of chelerythrine and protopine was the highest in the extract obtained after flowering.

### 2.2. Determination of Acetylcholinesterase Inhibition Activity of Alkaloid Standards

In the next step of the experiments the in vitro acetylcholinesterase inhibition activities of alkaloid standards: berberine, chelerythrine, chelidonine, protopine, and sanguinarine were examined. The determination of acetylcholinesterase inhibition activity was performed by the HPLC-DAD method. The chromatographic method for determination of this activity is much more accurate compared to the spectrophotometric method, especially for compounds such as berberine, sanguinarine, or chelerythrine, which show very high adsorption at the wavelength of 412 nm at which the determination of 5-thio-2-nitro-benzoic acid, a product of thiocholine, the product of the hydrolysis of acetylthiocholine reaction with Ellman reagent is usually performed. In the spectrophotometric method, it is not possible to prepare an appropriate reference solution, because the acetylcholinesterase inhibitors react and their concentrations are reduced, while in the case of the chromatographic method, only 5-thio-2-nitro-benzoic acid can be selective determined.

Investigations of acetylcholinesterase inhibition activity were performed after addition of 5, 25, and 125 µL of alkaloid standard solutions at concentration 1 mg/mL. Concentrations of alkaloid standards in final solutions were 10, 50, and 250 µg mL^−1^, respectively. Results were presented as % inhibition of acetylcholinesterase activity (Figure 1). After addition of 5 µL of alkaloids standards inhibition activities were very low except berberine (about 25% inhibition). Furthermore, after the addition of 25 µL of alkaloid standards, the inhibition was not too high. For this concentration, berberine also showed the highest activity. While, very high inhibition activity was observed for most alkaloids after the addition of 125 µL of their standards. The lowest activity was determined for protopine (only about 22% of inhibition), while the other alkaloids reduced the activity of acetylcholinesterase more than 40%. Chelerythrine and chelidonine caused 100% inhibition of acetylcholinesterase activity.

IC_50_ values for investigated isoquinoline alkaloid standards were also determined (Table 2). The weakest acetylcholinesterase inhibitory activities were obtained for protopine (IC_50_ = 69.81 µM), the highest for berberine and chelerythrine. The IC_50_ value for chelerythrine was 0.25 µg/mL (0.72 µM) and for berberine it was 0.36 µg/mL (1.06 µM). High acetylcholinesterase inhibition activity was also observed for sanguinarine. The IC_50_ for the alkaloid determined by the HPLC method was 1.85 µg/mL (5.57 µM).

### 2.3. Determination of Acetylcholinesterase Inhibition Activity Synergy of Pairs of Alkaloid Standards

**Figure 2:**
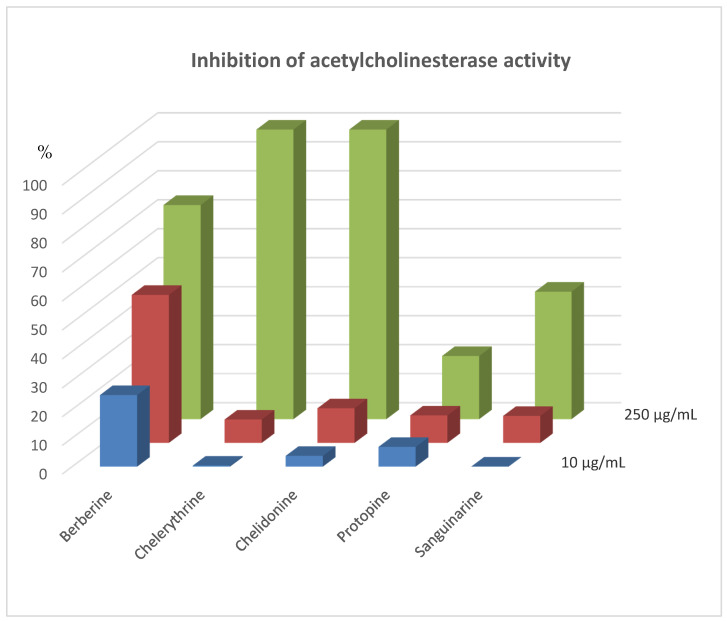
% inhibition of acetylcholinesterase activity after application of alkaloid standards at concentrations 10, 50, and 250 µg/mL.

**Figure 3:**
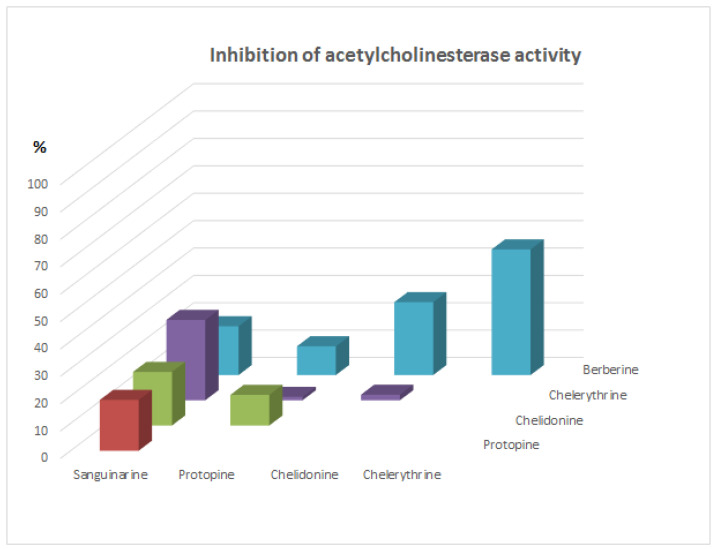
% inhibition of acetylcholinesterase activity after application of pairs of alkaloid standards at concentration 10 µg/mL.

Since these alkaloids occur together in plant extracts, the synergy of the inhibition of alkaloids was also investigated (Figure 2 and Figure 3). After addition of a mixture of two alkaloids at concentration 5 µg/mL, no significant increase in the inhibition of acetylcholinesterase activity was obtained for any pairs of investigated alkaloids.

While, a very significant synergism of the inhibition activity for all investigated alkaloids was observed after application of 25 µL of a mixture of two alkaloids containing 12.5 µL of each of them. The inhibition percent for each pair of alkaloids was very high, and was over 85%. The lowest activity was observed for the mixture of protopine with chelerythrine (about 87%), while the highest was the mixture of chelidonine with berberine (over 99%). Very high activity was also obtained for pairs: chelidonine—chelerythrine (98.8%) and protopine—sanguinarine (97.4%). Application of pairs of alkaloids in combinations after addition of 25 µL showed better acetylcholinesterase inhibition compared to the doses of single alkaloids in all cases.

The synergy was significantly higher when a mixture of all investigated alkaloids was applied to inhibition of acetylcholinesterase. After the addition of 5 µL of the mixture the inhibition was about 36%, while after the addition of 25 µL of the alkaloid mixture the 5-thio-2-nitro-benzoic acid peak was not identified which indicates practically 100% inhibition.

### 2.4. Determination of Acetylcholinesterase Inhibition Activity of Plant Extracts

The synergy effect is important for inhibition of acetylcholinesterase activity by plant extracts. Extracts obtained from *Sanguinaria canadensis* contained high concentrations of two isoquinoline alkaloids, sanguinarine and chelerythrine, and lower concentrations of other alkaloids with high acetylcholinesterase inhibition activity.

**Figure 4:**
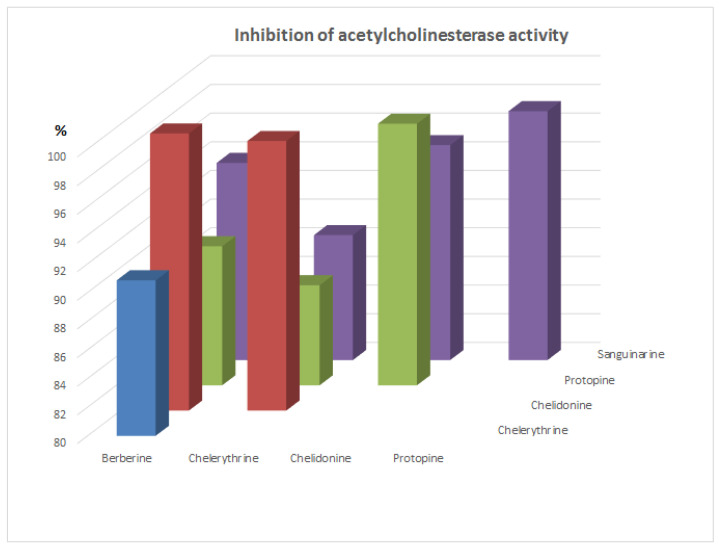
% inhibition of acetylcholinesterase activity after application of pairs of alkaloid standards at concentration 50 µg/mL.

**Figure 5:**
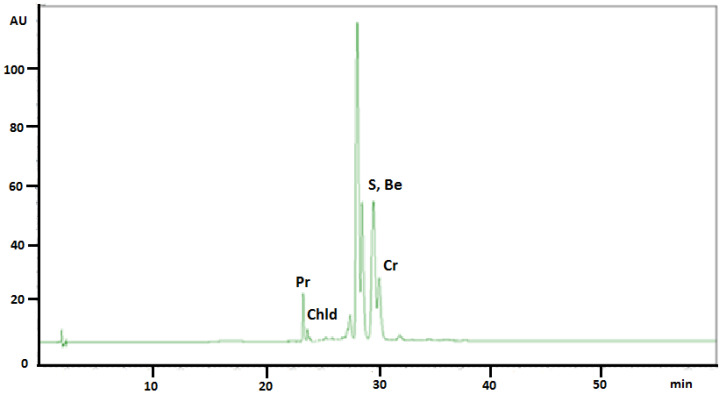
Chromatogram obtained on Polar RP column with mobile phase containing methanol, acetate buffer at pH 3.8, and 0.0025 M diethylamine in gradient mode (for details see Section 3) for extract obtained from *Sanguinaria canadensis* collected during flowering.

**Figure 6:**
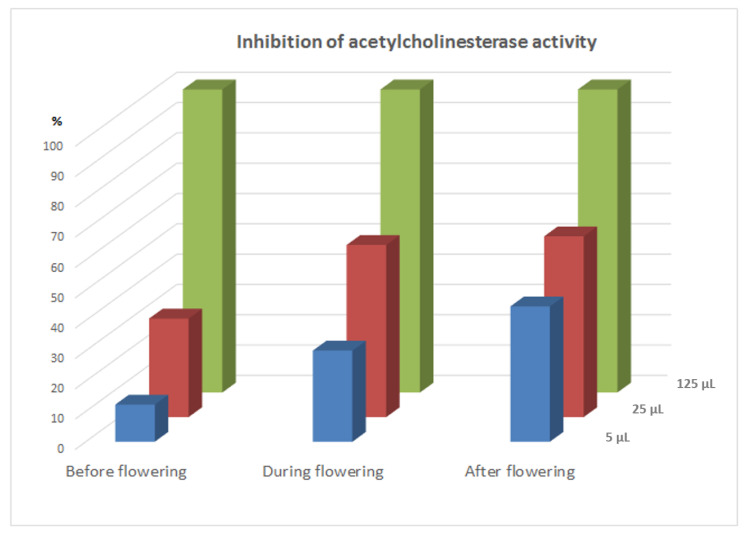
% inhibition of acetylcholinesterase activity after application of 10, 50, or 250 µg/mL solutions of *Sanguinaria canadensis* extracts obtained before, during, and after flowering.

In the next step of the experiments the acetylcholinesterase inhibition activity of extracts obtained from *Sanguinaria canadensis* collected before, during, and after flowering was investigated. Figure 4 presents a typical chromatogram obtained for extracts from *Sanguinaria canadensis* collected during flowering in a mobile phase system with addition of DEA applied for determination of 5-thio-2-nitro-benzoic acid which is the product of the reaction between the thiocholine and the Ellman reagent. The chromatogram obtained in the same chromatographic system for samples containing acetylcholinesterase after addition 15 µL *Sanguinaria canadensis* collected during flowering is presented in Figure 5. All tested extracts showed a high inhibition of acetylcholinesterase activity even after addition of 5 µL of 1 mg dry weight of extracts per milliliter (Figure 6). Inhibition was from 12% to over 44%. After the addition of plant extracts at concentration of 50 µg/mL, these values increased from 32% to almost 60%. The application of *Sanguinaria canadensis* extracts at concentration 250 µg/mL caused practically fully inhibition of acetylcholinesterase activity. The extract obtained from the plant before flowering exhibited the lowest activity (32% of inhibition after addition of plant extract at concentration 50 µg/mL). However, after the addition of this extract at concentration of 250 µg/mL the peak of 5-thio-2-nitro-benzoic acid was not identified on the chromatogram. A significant increase in the inhibition of acetylcholinesterase activity was observed after applying extracts obtained from *Sanguinaria canadensis* during flowering and after flowering. The acetylcholinesterase inhibition activities of these extracts were about 57% and 60%, respectively, after the application of both extracts at concentration of 50 µg/mL and increased to 100% after addition of these extracts at concentration of 250 µg/mL. The activity of acetylcholinesterase inhibition by *Sanguinaria canadensis* extracts correlates with the content of the determined isoquinoline alkaloids, but the activity of these extracts is certainly due to the presence of other compounds not determined by us. The chromatograms also contain peaks of substances that we have not identified, which may also affect their activity. This requires further research.

IC_50_ values obtained for *Sanguinaria canadensis* extracts are presented in Table 2. The highest acetylcholinesterase inhibition activity exhibit extract was obtained from *Sanguinaria canadensis* collected during flowering and the lowest extract was obtained from the plant collected before flowering.

## 3. Experimental

### 3.1. Apparatus and HPLC Conditions

Analysis was performed using an LC-20AD Shimadzu (Shimadzu Corporation, Canby, OR, USA) liquid chromatograph equipped with column Synergi polar RP 80A (150 mm × 4.6 mm, 5 μm). The chromatograph was equipped with a Shimadzu SPD-M20A detector (Shimadzu Corporation, Canby, OR, USA). Detection was carried out at a wavelength of 240 nm. All chromatographic measurements were controlled by a CTO-10ASVP thermostat (Shimadzu Corporation, Canby, OR, USA). The eluent flow rate was 1.0 mL/min. Extracts were injected into the columns using the Rheodyne 20 μL injector. The DAD detector was set in the 200–800 nm range. Data acquisition and processing were carried out with LabSolutions software (Shimadzu Corporation, Kyoto, Japan). The mobile phase was composed of 0.04 M 1-butyl-3-methylimidazolium tetrafluoroborate in water (solvent A) and 1-butyl-3-methylimidazolium tetrafluoroborate in acetonitrile (solvent B). The following gradient elution was applied: 0–20 min, 25% B; 20–30 min, 25–32% B; 30–40 min, 32–40% B, 40–60 min, 40% B. Flow rate was 1 mL/min [35].

### 3.2. Chemicals and Plant Materials

Acetonitrile (MeCN), methanol (MeOH), 1-butyl-3-methylimidazolium tetrafluoroborate, diethylamine, ammonium acetate, and acetic acid of chromatographic quality were obtained from E. Merck (Darmstadt, Germany). Acetylthiocholine iodide, 5,5′- dithiobis-(2-nitrobenzoic acid), and acetylcholinesterase were purchased from Sigma-Aldrich (St. Louis, MO, USA).

Alkaloid standards sanguinarine, chelerythrine, protopine, and chelidonine were purchased from Chem Faces Biochemical Co., Ltd. (Wuhan, China). Standard of berberine was purchased from Sigma-Aldrich (St. Louis, MO, USA).

Plant material was collected and identified in the Botanical Garden of Maria Curie-Skłodowska University in Lublin (Poland) in spring and summer 2020.

Plants were divided into roots and aboveground parts. Plant organs were cut into pieces and dried at ambient temperature for 1–2 weeks. Branches of *B. vulgaris* were decorticated and dried for 1–2 weeks under the same conditions.

### 3.3. Extraction Procedure

The previously described procedure of alkaloids extraction from plant material was applied after minor modifications [36]. Weighted samples (5 g) of each plant were macerated with 100 mL ethanol for 72 h and continuously extracted in an ultrasonic bath for 5 h. Extracts were filtered, the solvent evaporated under vacuum, and the residues dissolved in 30 mL of 2% sulfuric acid and defatted with diethylether (3 × 40 mL). The aqueous layers were subsequently basified with 25% ammonia to a pH of 9.5–10 and the alkaloids extracted with chloroform (3 × 50 mL). After evaporation of the organic solvent, the dried extracts were dissolved in 5 mL MeOH prior to HPLC analysis. Determination of berberine, protopine, and chelidonine was performed after dissolving dried extracts prepared in the same manner in 0.5 mL MeOH.

### 3.4. Determination of Acetylcholinesterase Inhibitory Activity

For determination of acetylcholinesterase inhibitory activity, HPLC analysis was performed on the same apparatus as determination of alkaloid contents on Polar RP column with mobile phase A containing methanol and 0.0025 M diethylamine, mobile phase B containing acetate buffer at pH 3.8 and 0.0025 M diethylamine, and mobile phase in gradient elution: 0–15 min, 10% A; 15–30 min, 10–70% A; 30–60 min, 70% A. Flow rate was 1 mL/min.

A stock solution of acetylcholinesterase at concentration 200 U/mL, acetylthiocholine iodide at concentration 0.0125 M and 5′-dithiobis-(2-nitrobenzoic acid) at concentration 0.00167 M were prepared. Samples for determination of acetylcholinesterase inhibitory activity were prepared by addition of 50 µL of acetylthiocholine iodide, 50 µL of 5′-dithiobis-(2-nitrobenzoic acid), 25 µL of acetylcholinesterase, and phosphate buffer at pH 7.8 to obtain 500 µL of final solution. The final concentration of acetylcholinesterase was 0.2 U/mL for determination of IC_50_ values and 10 U/mL for other investigations. All components were mixed and samples were incubated for 15 min at 37 °C. Samples were filtrated by syringe filter (CHROMAFIL Xtra, PVDF-29/25 0.20 µm) and injected into the HPLC system.

## 4. Conclusions

Extracts obtained from *Sanguinaria canadensis* collected before, during, and after flowering exhibited significant differences in the content of the investigated isoquinoline alkaloids. Very high contents of sanguinarine and chelerythrine as well as small amounts of other alkaloids were determined in all investigated extracts. The extract obtained from the plant collected before flowering contained the lowest amount of alkaloids. The content of all tested isoquinoline alkaloids increased significantly during the flowering of the plant, while after flowering the content of some alkaloids increased, while others decreased.

Most of investigated isoquinoline alkaloids exhibited a very high acetylcholinesterase inhibition activity. The IC_50_ obtained by our procedure for galantamine was 0.42 µg/mL (1.46 µM). Additionally, the investigated alkaloids demonstrated a synergistic effect. For all combinations of alkaloid pairs, inhibition of acetylcholinesterase activity was higher thann activity of single alkaloids at the same concentrations. The strongest synergy effect was observed for the mixture containing all investigated alkaloids.

All investigated extracts were found to be effective for acetylcholinesterase activity inhibition. Especially high activity was observed for extracts obtained from *Sanguinaria canadensis* collected during and after flowering. The extracts obtained from *Sanguinaria canadensis* are promising as inhibitors of acetylcholinesterase activity.

To the best of our knowledge, the acetylcholinesterase inhibition activity of *Sanguinaria canadensis* extracts before, during, and after flowering has not been investigated previously.

Ellman’s method using spectrophotometry for measurement of acetylcholinesterase activity is commonly applied for determination of acetylcholinesterase inhibition. However, this method has significant limitations when the measurement is carried out in solutions containing other substances that exhibit adsorption at a wavelength of about 412 nm, such as some alkaloids or many plant extracts. The application of the HPLC-DAD method for determination of acetylcholinesterase inhibition activity enables avoiding errors related to the overlapping adsorption of 5-thio-2-nitro-benzoic acid and some isoquinoline alkaloids occurring in extracts from *Sanguinaria canadensis*. In the literature there are only several reports on in vitro determination of acetylcholinesterase inhibition activity by chromatographic methods.

Based on these results, investigated plant extracts can be recommended for further in vivo experiments. Investigated extracts and alkaloids contained therein may be developed as a new candidate for treatment of neurodegenerative diseases such as Alzheimer’s disease.

## Figures and Tables

**Table 1:** Retention times and contents of isoquinoline alkaloids in plant extracts.

Alkaloid	Contents of Alkaloids in Plant Extracts Obtained from *Sanguinaria canadensis* mg/g of Dry Plant Material				Contents of Alkaloids in Plant Extracts Obtained from *Sanguinaria canadensis* µg/mg of Dry Plant Extract		
	t_R_	Before Flowering	During flowering	After Flowering	Before Flowering	During Flowering	After Flowering
Berberine (Be)	35.48	0.0058 (±0.0003)	0.0125 (±0.0010)	0.0091 (±0.0007)	0.46	0.80	0.61
Chelerythrine (Cr)	42.57	2.7224 (±0.0897)	5.3470 (±0.2018)	6.8722 (±0.1867)	216.41	341.88	459.68
Chelidonine (Chld)	19.33	-	<LOQ	-	-	<LOQ	-
Protopine (Pr)	14.18	<LOQ	0.0141 (±0.0008)	0.1075 (±0.009)	<LOQ	0.90	7.19
Sanguinarine (S)	36.82	4.8543 (±0.1207)	9.5899 (±0.2302)	6.9195 (±0.1624)	385.87	613.16	462.84

-: peak was not identified; <LOQ: below limit of quantification.

**Table 2:** IC_50_ values obtained for alkaloid standards and plant extracts (concentration of acetylcholinesterase 0.2 U/mL).

Alkaloid	Concentration of Acetylcholinesterase 0.2 U/mL	
	IC_50_ (µg/mL)	IC_50_ (µM)
Berberine	0.36 (±0.03)	1.06
Chelerythrie	0.25 (±0.02)	0.72
Chelidonine	9.53 (±0.43)	26.97
Protopine	23.13 (±0.91)	69.81
Sanguinarie	1.85 (±0.12)	5.57
***Sanguinaria canadensis* Extracts**		
Before flowering	89.14 (±4.03)	-
During flowering	61.24 (±3.12)	-
After flowering	64.12 (±3.34)	-

-: the value cannot be calculated for extract.

## Data Availability

Not applicable.
